# Seronegative Autoimmune Hepatitis Following Coronavirus Disease 2019 (COVID-19) Infection

**DOI:** 10.7759/cureus.79068

**Published:** 2025-02-15

**Authors:** Mohammad Kloub, Khaled A Elfert, Flor Rosado, Ahmed Elnajjar, Mohamed Eldesouki, Abdul-Rahman I Abusalim

**Affiliations:** 1 Department of Internal Medicine, Saint Michael's Medical Center, New York Medical College, Newark, USA; 2 Department of Internal Medicine, St. Barnabas Hospital Health System, New York, USA; 3 Department of Gastroenterology, Al-Shifa Hospital, Gaza, PSE; 4 Department of Internal Medicine, University of Wisconsin School of Medicine and Public Health, Madison, USA

**Keywords:** autoimmune hepatitis (aih), case report, clinical autoimmunity, covid-19 infection, molecular mimicry

## Abstract

Autoimmune hepatitis is an autoimmune liver condition of uncertain etiology. Environmental triggers have been involved in the pathophysiology of the disease. The triggers include viruses, immunizations, and drugs. Since the emergence of the coronavirus disease 2019 (COVID-19) pandemic, severe acute respiratory syndrome coronavirus 2 (SARS-CoV-2) has been implicated in the development of various autoimmune diseases. We report the case of a 58-year-old patient who had persistent elevation in liver transaminase levels after COVID-19 infection. After undergoing a liver biopsy, he was diagnosed with seronegative autoimmune hepatitis with an excellent response to steroids. Our case highlights the importance of considering the diagnosis of autoimmune hepatitis in patients with persistent elevation of liver transaminases after COVID-19 infection.

## Introduction

Autoimmune hepatitis (AIH) is an autoimmune liver condition that occurs predominantly in females and can present at any age. Different environmental triggers have been thought to contribute to the development of AIH in genetically predisposed individuals. These triggers include viruses, medications, and vaccinations [1,2]. The clinical presentation of AIH is variable and includes acute liver failure, chronic liver disease, asymptomatic elevation of liver function tests (LFTs), and autoantibody-negative hepatitis [3]. Diagnosis relies on characteristic serological and histological markers and excludes other causes of liver disease. 

The coronavirus disease 2019 (COVID-19) infection has been linked to the production of autoimmune antibodies and the development of autoimmune diseases [4]. Here, we report a patient who developed a COVID-19 infection and was subsequently diagnosed with probable seronegative AIH. This case adds to the few published cases linking COVID-19 infection to the development of AIH. Also, this is the second case of seronegative AIH after COVID-19 infection [5].

## Case presentation

**Figure 1:**
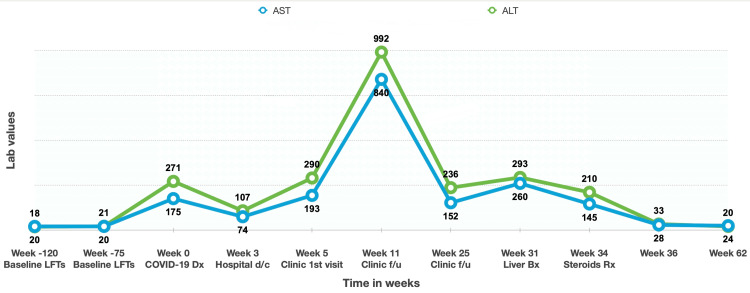
Trend of liver enzymes over time ALT: alanine aminotransferase; AST: aspartate aminotransferase; Bx: biopsy; d/c: discharge; Dx: diagnosis; f/u: follow-up; LFTs: liver enzymes including AST and ALT; Rx: treatment

A 58-year-old Egyptian male patient, known to have vitiligo and mild rheumatic mitral stenosis, presented to Hamad General Hospital in Qatar in September 2020 complaining of flu-like symptoms. The patient denied any history of alcohol intake, substance abuse, over-the-counter or herbal medications, or a family history of liver or autoimmune disease. His chronic medications included aspirin and bisoprolol. His clinical examination was unremarkable. The COVID-19 polymerase chain reaction (PCR) test was positive, so he was admitted for observation. Chest X-ray showed no abnormality, and he was not started on any medication. His laboratory investigations were remarkable for alanine aminotransferase (ALT) of 271 and aspartate aminotransferase (AST) of 175 U/L (Figure 1). During hospitalization, liver enzymes were trending down but remained elevated. He did not receive antibiotics or antivirals during his hospital stay.

His biochemical testing showed positive hepatitis B virus (HBV) surface antibody and HBV core IgG antibody, with negative HBV surface antigen and HBV PCR indicating past infection. Hepatitis E virus (HEV) antibody (Ab) serology was positive for IgG Ab and negative for IgM Ab, consistent with prior infection. Other viral hepatitis work-ups revealed negative hepatitis A, hepatitis C, parvovirus B19, and herpes simplex viral serologies. Cytomegalovirus and Epstein-Barr viral PCR were negative as well.

The patient underwent additional laboratory work-up for other etiologies of liver disease that revealed normal ceruloplasmin and alpha-1 anti-trypsin. His AIH work-up showed that the antinuclear antibody (Ab) screening test, which was conducted using fluoroenzyme immunoassay, was negative, and it was confirmed by an indirect immunofluorescent technique using a human epithelial type 2 (HEP-2) cell line. Other autoimmune work-ups revealed negative anti-mitochondrial M2 Ab, anti-smooth muscle Ab, and anti-liver kidney microsome Ab testing. He had an elevated IgG level of 16.9 g/l.

His radiological investigations included an abdominal ultrasound that revealed mildly increased echotexture, a liver magnetic resonance imaging (MRI) that was only remarkable for a tiny cyst of 2 mm, and liver elastography that showed a stiffness average of 5.77 kPa.

**Figure 2:**
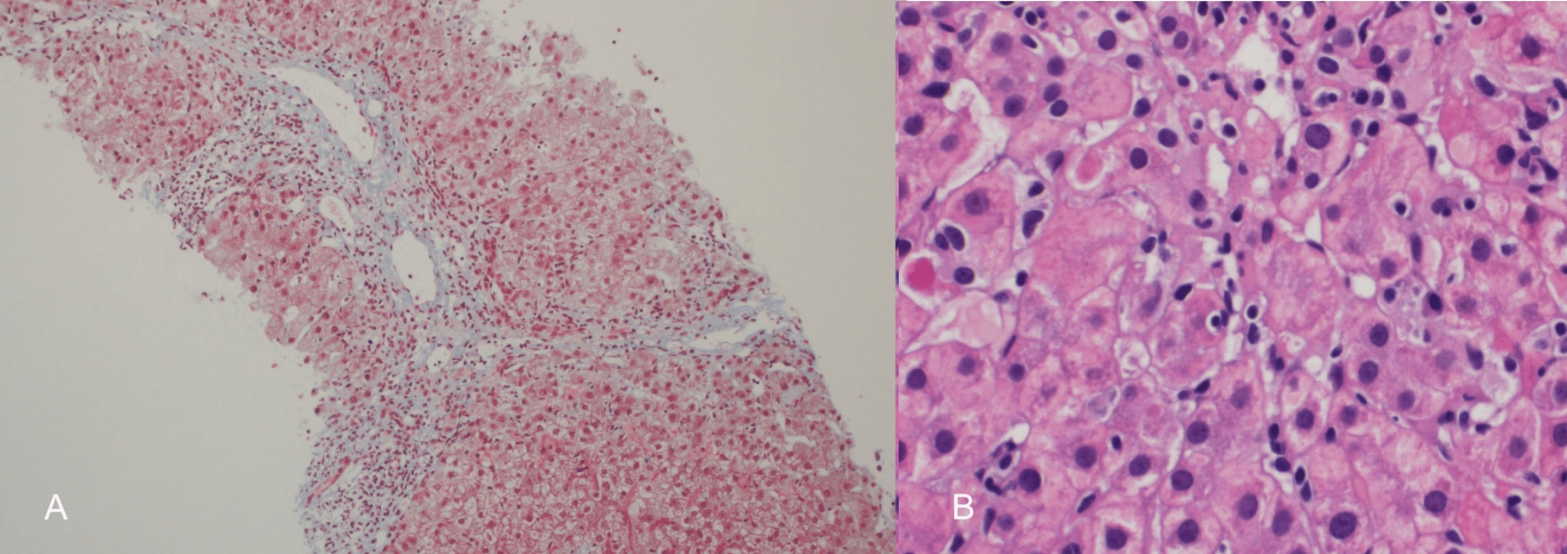
Liver biopsy (A, B) Histopathology picture showing moderate interface and portal tract inflammatory cell infiltrate composed mainly of lymphocytes with occasional eosinophils

Initially, a liver biopsy was recommended, but the patient opted to defer the procedure. He consented to the procedure on week 31, given the persistent elevation of the LFTs (Figure 1). The liver biopsy showed moderate interface and portal tract inflammatory cell infiltrate composed mainly of lymphocytes with occasional eosinophils. Bile ducts were unremarkable, and there was no significant steatosis or iron deposition (Figure 2). Based on the biopsy result, the pre-treatment revised original scoring system for AIH was 14, suggesting probable AIH (Table 1). On week 34, the patient was started on prednisone 40 mg. LFTs on week 36 were completely normal. The prednisone was tapered over the following two months. Six months later, repeat LFTs were also normal (Figure 1).

**Table 1:** The revised original scoring system for AIH and its applications to our patient ALP: alkaline phosphatase; AST: aspartate aminotransferase; ALT: alanine aminotransferase; ANA: antinuclear antibody; SMA: smooth muscle antibody; LKM-1: liver kidney microsome type 1 antibody; AMA: anti-mitochondrial antibody; AIH: autoimmune hepatitis

Parameters/features	Reference score	Patient score
Female sex	+2	0
ALP:AST (or ALT) ratio		
<1.5	+2	+2
1.5-3.0	0	0
>3.0	−2	0
Serum globulins or IgG above normal		
>2.0	+3	0
1.5-2.0	+2	0
1.0-1.5	+1	+1
<1.0	0	0
ANA, SMA, or LKM-1		
>1:80	+3	0
1:80	+2	0
1:40	+1	0
<1:40	0	0
AMA positive	−4	0
Hepatitis viral markers		
Positive	−3	0
Negative	+3	+3
Drug history		
Positive	−4	0
Negative	+1	+1
Average alcohol intake		
<25 g/day	+2	+2
>60 g/day	−2	0
Liver histology		
Interface hepatitis	+3	+3
Predominantly lymphoplasmacytic infiltrate	+1	0
Rosetting of liver cells	+1	0
None of the above	−5	0
Biliary changes	−3	0
Other changes	−3	0
Other autoimmune disease(s)	+2	+2
Optional additional parameters		
Seropositivity for other defined autoantibodies	+2	0
HLA DR3 or DR4	+1	0
Response to therapy		
Complete	+2	+2
Relapse	+3	0
Interpretation of aggregate scores (pre-treatment)		
Definite AIH	>15	-
Probable AIH	10-15	14
Interpretation of aggregate scores (post-treatment)		
Definite AIH	>17	-
Probable AIH	12-17	16

## Discussion

The patient presented with COVID-19 infection and subsequently developed persistent elevation in his liver enzymes, requiring extensive work-up. At first, it was thought that the rise of LFTs could be attributed to the COVID-19 infection, considering the link between the viral infection and LFTs' elevation [6]. However, this assumption was refuted by the fact that the LFTs remained elevated for 34 weeks. The patient had a BMI of 41.5, which suggested the possibility of non-alcoholic steatohepatitis (NASH). NASH diagnosis was excluded as LFTs had been normal for two years prior to his admission (Figure 1) despite maintaining a consistent weight of 117-120 kg from 2016 to 2020. Also, LFTs remained within the normal range for nine months after starting steroid treatment, even with the high BMI. Moreover, the liver biopsy didn't show significant steatosis. The laboratory testing showed positive hepatitis viral serology for HBV core IgG Ab, HBV surface Ab, and HEV IgG antibody, indicating past hepatitis B and E infection. The persistent elevation in LFTs for months couldn't be explained by this positive serology, given the negative HBV surface Ag and HEV IgM Ab during the initial work ruling out the presence of an active hepatitis B or E infection. We used the revised original scoring system for AIH as a diagnostic tool, given its superiority in patients manifesting with few or atypical features [7]. Table 1 describes the different components of the scoring system and its application to our patients. Our patient's post-treatment score was 16, indicating probable AIH.

Knight et al. described how severe acute respiratory syndrome coronavirus 2 (SARS-CoV-2) shares characteristics with other viruses that also trigger autoimmunity in predisposed patients. These viruses include cytomegalovirus, parvovirus B19, and Epstein-Barr virus. The shared characteristic features include the precedence of viral infection to autoimmune diseases, molecular and functional mimicry, induction of robust type I interferon (IFN) responses in predisposed patients, autoantibody production (breaking the host's immune tolerance), and possessing superantigen-producing activity [8]. These features support the theory of de novo autoimmunity triggered by COVID-19 infection. Moreover, it has been reported that COVID-19 infection is associated with the development of autoimmune diseases, such as Guillain-Barré syndrome, systemic lupus erythematosus, autoimmune hemolytic anemia, and type 1 diabetes mellitus (DM) [4]. This further supports the theory that COVID-19 is linked to the development of AIH.

Immunological reactions and autoimmune diseases may be connected to molecular mimicry secondary to the cross-reactivity of the spike protein of SARS-CoV-2 with human tissue due to viral-antigenic mimicry and bystander activation, which is characterized by the autoreactive stimulation of immune cells, mainly CD8+ T cells, following antigen-specific responses against the viral-specific antigens. The proliferation of CD8+ T cells can be mediated by interactions of interleukin-15 with its receptor on the surface of the antigen-presenting cell, which can initiate the bystander stimulation of memory-phenotype CD8+ T cells [9,10]. Furthermore, protein analysis showed a significant similarity between a human protein (titin) and the SARS-CoV-2 spike glycoprotein. The peptide sharing between the two species may trigger immunological reactions that cross-react with human proteins, leading to autoimmune disease [11].

Although the case presents a correlation between COVID-19 and subsequent probable AIH development, it is important to understand that this doesn't establish a direct causal relationship. Upon literature review, we found few cases in which COVID-19 infection was linked to AIH [5,12-16]. Similarly, the association of AIH with COVID-19 vaccines has been widely documented, with molecular mimicry considered one of the most commonly proposed mechanisms [17]. Interestingly, in a report of 32 cases of COVID-19 vaccine-induced AIH, 31.3% of the cases had completely negative autoantibody panels, similar to our patient [18]. In conclusion, clinicians should consider the diagnosis of AIH in patients with persistent elevation of liver transaminases after COVID-19 infection.

## Conclusions

AIH is an autoimmune liver condition of uncertain etiology that occurs predominantly in females and can present at any age. This case report highlighted the importance of considering the diagnosis of AIH in patients with persistent elevation of liver transaminases after COVID-19 infection. Early recognition and treatment are crucial to prevent complications.
